# Flexible bronchoscopy in a tertiary healthcare facility: a review of indications and outcomes

**DOI:** 10.4314/gmj.v55i1.4

**Published:** 2021-03

**Authors:** Jane S Afriyie-Mensah, Ernest Kwarteng, John Tetteh, Lawrence Sereboe, Audrey Forson

**Affiliations:** 1 Department of Medicine and Therapeutics, University of Ghana Medical School, Legon, Accra, Ghana; 2 Research Department of the University of Ghana Medical School, Accra, Ghana; 3 National Cardiothoracic Centre, Korle-Bu Teaching Hospital, Korle-Bu, Accra, Ghana; 4 Department of Community Health, University of Ghana Medical School, College of Health Sciences, University of Ghana, Accra, Ghana

**Keywords:** Bronchoscopy, Bronchoalveolar lavage, Pulmonary Tuberculosis

## Abstract

**Objectives:**

Flexible Fibreoptic bronchoscopy (FFB) is a major diagnostic and therapeutic tool employed largely in respiratory medicine but its use in our country has been quite limited. We performed a retrospective review of the indications, overall diagnostic yield and safety of FFB at the Korle-Bu Teaching Hospital (KBTH).

**Study Design:**

Retrospective study

**Study Setting:**

Cardiothoracic Unit, Korle-Bu Teaching Hospital

**Study Participants:**

All bronchoscopy records from January 2017 - December 2018

**Interventions:**

Eight-five bronchoscopy reports generated over a 2-year period were reviewed. Using a data extraction form, patient's demographic details, indications for FFB, sedation given, specimen obtained and results of investigation, and complications encountered were recorded and entered into SPSS version 22. Descriptive analysis was performed and presented as means and percentages.

**Results:**

Suspected lung cancer was the predominant indication for bronchoscopy requests (55.3%). Diagnostic yield of endobronchial biopsy was 86.7% increased to 93.3% when biopsy was combined with bronchial washing cytology. Bronchial washing geneXpert was positive in 20.8% of sputum negative cases, and 20.7% of patients with unresolved pneumonia and bronchiectasis had a positive microbial yield. Overall mild complications occurred in 5.9% of patients with no mortality.

**Conclusion:**

Flexible bronchoscopy has a significantly high diagnostic yield, particularly in evaluating lung cancers and undiagnosed lung infections with minimal associated complications, hence increasing its availability in the country and widening the diagnostic scope at the cardiothoracic unit of the Korle-Bu Teaching Hospital.

**Funding:**

None declared

## Introduction

Flexible fibreoptic bronchoscopy (FFB) is an important medical procedure that involves the direct visualization of the tracheobronchial tree using a fibreoptic scope and is pivotal in diagnosing a variety of respiratory diseases, particularly lung cancer.^1^ Flexible bronchoscopy has improved from the traditional rigid bronchoscopy, which has a limited scope of visualizing only the central airways unless combined with the versatile, flexible bronchoscope.^2^ Rigid bronchoscopy is a more invasive procedure performed under general anaesthesia whiles FFB is mostly performed as a day case with conscious sedation. The latter affords much patient comfort and a thorough examination up to the peripheral airways.^2^

It is a fairly safe investigative tool in the hands of the experienced pulmonologist/thoracic surgeon with minimal complications although could rarely be life-threatening.^1^ Some major indications of FFB includes evaluating clinical symptoms such as hemoptysis, hoarseness of voice, stridor, persistent cough and/or radiological findings including suspected centrally located lung masses, suspected bronchial obstruction (as may underlie recurrent pneumonia and persistent lung collapse), unresolving pneumonia and bronchiectasis.^3^ Evaluating interstitial lung diseases, inhalational injury, suspected bronchopleural or trachea-esophageal fistulae are other known indications of bronchoscopy.^1^,^3^ Therapeutic interventions of FFB include removing foreign bodies, blood clots and impacted mucus in the airways.^1^,^3^

Advancement in technology has led to new therapeutic measures such as stent placement in airway obstruction, lung volume reduction in Chronic Obstructive Pulmonary Disease (COPD) and bronchial thermoplasty in difficult asthma.^3^ The most phenomenal addition is endobronchial ultrasound (EBUS) which makes mediastinal masses, including lymph nodes and pulmonary nodules, accessible via FFB.^4^. Also, navigational bronchoscopy and cryobiopsies have enhanced the evaluation of peripheral pulmonary nodules and interstitial lung diseases respectively.^5^

Similar to the situation in many resource-challenged African countries,^6^ the availability of FFB as well as the skilled human resources is limited in our country being performed only at the premier tertiary hospital, where mainly traditional diagnostic techniques are employed (endobronchial biopsy and collection of respiratory secretions by either bronchoalveolar lavage or bronchial washing). The limited availability and the narrow scope of bronchoscopy in the country presents a major setback in the early and appropriate diagnosis of respiratory conditions, particularly lung cancers and interstitial lung diseases, subsequently affecting their management. Although the FFB service at the cardiothoracic unit was set up about ten years ago, it recorded only about 3–5 bronchoscopy cases annually due to the lack of permanent skilled personnel. However, with pulmonologists currently at the premier tertiary facility, the practice has seen a significant increase in the number of bronchoscopy requests over the last 3–4 years with a growing need for improved diagnostic and therapeutic pulmonary interventions. Referrals to the unit are received from other health facilities in the country but largely from within the hospital, particularly the Respiratory clinic and Radiology units. The development of a robust practice with guiding protocols as well as an enhanced skill set of practicing pulmonologists is therefore urgently required.

As per British Thoracic Society (BTS) recommendations,^7^ we undertook a review of cases performed over the past 2-year period with regards to indication for bronchoscopy requests, bronchoscopic findings, endobronchial sampling techniques employed and its diagnostic yield as well as the complications encountered. Results of this review will help evaluate the practice procedure of bronchoscopy at the Korle-Bu Teaching Hospital as per recommended guidelines, its diagnostic yield and identify measures needed to promote best practice. This will ultimately guide the development of an expanded bronchoscopy service in the immediate future.

## Methods

We retrospectively reviewed the records of 98 patients who had FFB between the periods of January 2017 to December 2019 at the endoscopy suite of the cardiothoracic unit of KBTH using an Olympus EVIS EXERA II CLV-160 bronchoscope. The bronchoscopy suite is not a negative pressure room hence all procedures are usually done with surgical masks and N95 masks for suspected lung infections especially Pulmonary Tuberculosis (PTB). Bronchoscopy procedures were performed on scheduled and pre-procedure fasted patients with INR ≤ 1.5. Lignocaine spray (2%) was used as topical anaesthetic agent for numbing the oropharyngeal region and intravenous midazolam, atropine and fentanyl as premedication agents either singly or in combination.^7^ Patients routinely received supplementary oxygen via nasal prongs with continuous monitoring of vitals (SpO2, heart rate and blood pressure).

Relevant data extracted from the bronchoscopy records using a data extraction tool included; patient demographics, indication for the procedure, coagulation screen (INR), patients' blood pressure, pulse rate and oxygen saturation (pre and post bronchoscopy), medications used for sedation and bronchoscopy findings. Histology, cytology, microscopy/culture and sensitivity, and GeneXpert results available in the bronchoscopy folders were also extracted. The outcome of the procedure was assessed based on the diagnostic yield of samples obtained, and complications encountered such as hypoxaemia, hypotension, significant bleeding requiring intervention or treatment and death.

Descriptive analysis was performed using SPSS version 22 to achieve study objectives. Ethical clearance was obtained from the University of Ghana College of Health Sciences, Ethical and Protocol review committee with an ID number of CHS-Et/M2-5.2/2019-2020

### Study Definitions

- The diagnostic yield of a tissue sampling technique was defined as the proportion of positive results obtained when the bronchial sample was subjected to the appropriate laboratory investigations
- Complications related to the procedure was defined as any bronchoscopy-related adverse effect that required an intervention/management during or immediately after bronchoscopy

## Results

Thirteen bronchoscopy reports had incomplete data due to ineligible information on the carbon-copy report sheets and unavailable laboratory results, thus 85 case reports were captured and included in the analysis.

All bronchoscopy procedures performed over the two years were for diagnostic purposes. These included 38(44.7%) females and 47(55.3%) males. The predominant age group among scoped patients was 40–59 years (41.2%), with a mean of 53.9(±14.8) years (Table 1).

**Table 1:** Demographic characteristics, presenting symptoms and indications for bronchoscopy

Variable	Frequency n(%)
**Age group [Mean(SD)]**	[53.89(14.76)]
**≤ 39**	17(20)
**40–59**	35(41.2)
**60+**	33(38.8)
**Sex**	
**Female**	38(44.7)
**Male**	47(55.3)
**Symptoms**	
**Cough**	
**Yes**	67(78.8)
**No**	18(21.2)
**Weight loss**	
**Yes**	38(44.7)
**No**	47(55.3)
**Hemoptysis**	
**Yes**	20(23.5)
**No**	65(76.5)
**Shortness of Breath**	
**Yes**	32(37.6)
**No**	53(62.4)
**Indications for bronchoscopy**	
**Lung Cancer**	47(55.3)
**Hemoptysis**	1(1.2)
**PTB**	9(10.6)
**Tracheal tumor**	4(4.7)
**Unresolving pneumonia and bronchiectasis**	9(10.6)
**Fistula**	2(2.4)
**Sarcoidosis**	3(3.5)
**Pleural effusion**	3(3.5)
**Lung collapse**	7 (8.2)

### Indications for bronchoscopy

The most prevalent indication for bronchoscopy was suspected lung cancer 47(55.3%). Other indications included suspected pulmonary TB 9(10.6%), unresolved pneumonia and bronchiectasis 9(10.6%), tracheal tumors 4(4.7%), investigation of lung collapse 7(8.2%), pleural effusion 3(3.5%), suspected tracheoesophageal fistula 2(2.4%), pulmonary sarcoidosis 3(3.5%) and hemoptysis 1(1.2). The predominant respiratory symptom recorded among the cases scoped was cough (78.8%) (Table 1).

### Pre-procedure sedation and coagulation screen for bronchoscopy

Except for two patients, the remaining 83 (97.6%) had pre-medication via the intravenous route. Forty-three (51.8%) received only Midazolam, 18(21.7%) had a combination of Midazolam and Fentanyl, 19(22.9%) had both Midazolam and IV Atropine and 3(3.6%) received all three medications. Most of the patients 75(90.3%) had 1–2mg of Midazolam with the rest receiving 3mg. Of those who had fentanyl, 12(57.1%) had a dose of 25mcg, and 9(42.8%) had 50mcg. The atropine dose was 0.5mg for all. The mean INR obtained before the procedure was 1.25 with a range of 0.9–1.6 (Table 2).

**Table 2:** Types of premedication agents administered, bronchoscopy findings and outcomes

Variable	Frequency N(%)
**Pre-medication**	
**Midazolam**	
**Yes**	83(97.6)
**No**	2(2.4)
**Fentanyl**	
**Yes**	21(24.7)
**No**	64(75.3)
**Atropine**	
**Yes**	22(25.9)
**No**	63(74.1)
**Combined medications given (N=83)**	
**Midazolam only**	43(51.8)
**Midazolam + Fentanyl**	18(21.7)
**Midazolam + Atropine**	19(22.9)
**Midazolam + Fentanyl+ Atropine**	3(3.6)
**Bronchoscopy findings (N=83)**	
**Endobronchial Tumor**	26(31.3)
**Indurated/Erythematous Mucosa**	25(30.1)
**Tracheal Mass**	3(3.6)
**Normal**	29(34.9)
**Specimen taken**	
**Forceps biopsy**	30(36.1)
**Bronchial wash**	65(78.3)
**Complications encountered**	
**Nil**	80(94.1)
**Bleeding**	3(3.5)
**Broncho/Laryngospasm**	2(2.4)
**Histology report (N=26)**	
**Adenocarcinoma**	14(53.8)
**Squamous cell carcinoma**	3(11.5)
**Bronchial carcinoids**	4(15.4)
**Atypical adenomatous hyperplasia**	3(11.5)
**Adenoid cystic carcinoma**	2(7.7)

### Bronchoscopy findings, specimen taken and diagnostic yield

Bronchoscopy findings were analyzed in 83 cases as two could not tolerate the procedure shortly after intubation past the vocal cords. Twenty-nine (34.9%) had endoscopy visible tumours (26 endobronchial and three tracheal), 25(30.1%) had erythematous, or indurated mucosa or post-inflammatory mucosal changes at various points of the bronchial tree with varying severity and 29(34.9%) had normal bronchoscopy findings. All visible endoscopy tumours and a couple of significantly indurated mucosal lesions were sampled using the forceps biopsy needle except for one tracheal tumour found to be unsuitable for biopsy (significantly vascularized tumour).

Bronchial washings were obtained from 65 patients including all those with abnormal bronchoscopy findings and others with suspected lung infections (Table 1). Out of the 30 endobronchial biopsies sent for histopathology, 26 were diagnostic of malignancy giving a diagnostic rate of 86.7%. The histopathology diagnoses were adenocarcinoma 14(53.8%), squamous cell carcinoma 3(11.5%), bronchial carcinoids 4(15.4%), atypical adenomatous hyperplasia 3(11.5%) and adenoid cystic carcinoma of trachea 2(7.7%). The four non-diagnostic tissue biopsies (13.3%) were a result of inadequate sample. Two out of the four negative histopathology reports had malignant cells on cytology of bronchial washings giving a malignancy pick-up rate of 93.3% when biopsy was combined with bronchial washing cytology. In total bronchial washing cytology alone was suggestive of malignancy in 21 samples giving a diagnostic rate of 70%. No brush or transbronchial biopsies were obtained.

A total of 24 BW samples from cases with suspected pulmonary infections were sent for GeneXpert and 5 (20.8%) had mycobacterium bacilli detected. A total of 29 BW samples were sent for microscopy, culture and sensitivity with positive microbial culture in 6(20.7%) samples. From the analysis, 28(59.6%) out of the 47 patients with suspected lung cancer had suggestive histology and/or cytology. All seven cases with suspected lung collapse scoped had inflammatory or post-inflammatory mucosal changes with none having an endobronchial tumor.

### Complications

Three (3.5%) patients had bleeding associated with endobronchial forceps biopsy that required instillation of cold saline and/or adrenalin with no sequelae. Two (2.4%) patients had bronchospasm leading to cancellation of procedure and required nebulization with a bronchodilator. There were no records of hypotension, persistent hypoxaemia, arrhythmia or deaths encountered over the study period. The overall complication rate of FFB encountered was 5.9%.

## Discussion

The first commercially available flexible bronchoscope was introduced by Dr. Shigeto Ikeda in 1967, which allowed visualization and sampling of the airway up to the fourth order subsegmental bronchi.^8^ An improved form of the flexible scope led to the introduction of the video bronchoscope in 1987 which projects images on a video monitor for educational purposes and stored for future reference.^9^

The usefulness of FFB in the evaluation of respiratory diseases cannot be over emphasized but has limited availability in resource poor countries with little data from most African countries. The recent significant increase in bronchoscopy requests observed at the CTU is encouraging and likely due to the increased awareness of this diagnostic tool among clinicians and the availability of pulmonologists. From the analyses, evaluation for airway malignancies (lung cancer and tracheal tumors) together constituted 60% of all bronchoscopy requests not including those with an indication of lung collapse.

This figure was much higher compared to 33% lung cancer indication reported in a Nigerian study but similar to that observed in a Tanzanian study (67.6%).^10^,^11^ The predominance of lung cancer as an indication depicts the importance of bronchoscopy in securing this diagnosis, particularly for central airway tumors, which are otherwise not amenable to percutaneous Ct-guided biopsy. The true prevalence of lung cancers in Africa, as well the histological subtypes, generally thought to be low or unknown, could therefore be a result of the limited availability of investigative procedures such as flexible bronchoscopy. Infectious causes of pulmonary disease were the next prevalent indication noted (24.7%) being similar to findings in a South African study with 30% attributable to pulmonary tuberculosis and non-resolving pneumonia^12^. Being in a TB endemic country,^13^ there is a high index of suspicion for pulmonary tuberculosis among patients with interstitial infiltrates on chest imaging, thus in the absence of a positive sputum GeneXpert, bronchoscopy with BAL/BW is required to clinch diagnosis of PTB or otherwise. Interestingly, all 7 cases of suspected lung collapse scoped at the unit had no endobronchial tumor but rather had inflammatory or post-inflammatory mucosal changes, 2 of whom had a previous history of PTB. On exploration of the tracheobronchial tree, no tracheoesophageal fistulae were detected in those with this indication. It was also noted that evaluation of interstitial lung diseases (ILDs) with FFB was barely requested for with only 3 cases of pulmonary sarcoidosis scoped at the CTU.

Biopsy of an endobronchial tumor is very crucial for investigating central lung tumors or polyps and has a good diagnostic accuracy when enough samples are taken for histopathology. Consistent with the major indication of FFB in this study, endoscopically visible airway tumors/lesions constituted 31/54(57.4%) of all abnormal bronchoscopy findings. This proportion is similar to 41(68.3%) and 330(55%) visible endobronchial lesions reported at bronchoscopy by Mahzar et al^14^ and Zavala et al^15^. Prior chest CT-scans has over the years improved patient selection for bronchoscopy as well as its diagnostic yield since endobronchial extension can be easily detected radiologically, thereby reducing the cost burden of investigations in patients suspected to have lung cancer.^16^

By making prior chest CT a pre-requisite for bronchoscopy at the CTU, a significant proportion of suspected lung cancer patients scoped 28/47(59.6%), actually had cancer confirmed on histology with sub-typing. Previous studies have estimated the diagnostic yield of endobronchial forceps biopsy to be between 65–82% with a further increase to 90% when combined with bronchial washings cytology and/or brush biopsy.^17^,^18^ The diagnostic rate of endobronchial biopsy in this study was comparatively higher (86.7%) with enhanced sensitivity (93.3%) when combined with BW cytology, with the later alone showing a much lower sensitivity of 70% in diagnosing Lung cancer. This emphasizes the importance of multiple sampling during bronchoscopy, being cost efficient also^19^. Although, combining endobronchial forceps and brush biopsies have been shown to be superior in terms of diagnostic yield,^19^ we performed no brush biopsies at the unit over the 2-year period due to unavailability of biopsy brushes. In spite of the absence, the diagnostic yield achieved with endobronchial forceps biopsy and BW was significantly high. This important observation could lead to adopting the latter combination as it is costly for the unit to procure biopsy brushes coupled with the increased cost of processing 3 samples in the pathology lab as patients pay out of pocket for these investigations. The predominant histological subtype of adenocarcinoma is consistent with current data which shows that it is the most prevalent subtype of lung cancers^20^. The presence of relatively rare subtypes in our cohort of patients such bronchial carcinoids (12%) and pre-invasive atypical adenomatous hyperplasia (12%) was significant.

Broncho-alveolar lavage (BAL) entails obtaining secre tions or fluid from the lower respiratory tract (smaller bronchi and alveoli) by flushing repeated aliquots of 20–60mls (total volume between 100–300mls) of normal saline into an affected lung area by wedging the bronchoscope between bronchi and suctioning back through the scope.^7^ Traditionally, the right middle lobe and the lingula are sampled due to adequate return of instilled fluid. BAL should be performed prior to any other bronchoscopic procedure such as endobronchial or transbronchial biopsy. The obtained lavage usually contains the cellular milieu of the alveoli (inflammatory cells/microorganisms) with minimal contamination from the larger airways.

It is therefore very essential in diagnosing interstitial lung diseases and lung infections. Bronchial washing (BW) is distinguished from BAL in that it samples fluid from the larger airways hence requires smaller volume of instilled normal saline (20ml) being easy and quick to perform and can be collected pre or post biopsy.^7^ Bronchial washing obtained after biopsy is best suited for cytological detection of malignant cells and sensitive for diagnosing lung infections such as bronchiectasis, recurrent pneumonias and tuberculosis.^7^,^21^ Its role in enhancing the diagnosis of PTB in at-risk patients with smear negative sputum samples has been shown to be significant.^2^ In the current study, BW for GeneXpert was diagnostic of PTB in 20.8% of suspected patients, a value comparable to findings in South Africa.^12^ A study by Patil et al in India, however, reported positive GeneXpert in 91.2% of 250 smear negative PTB cases.^21^

The cost of FFB at the CTU coupled with the low threshold of initiating anti-tuberculous medications in our clinics could explain the low bronchoscopy requests for suspected PTB seen at the unit and could explain the lower yield compared to the Indian study. Also, 6(20.7%) out of the 29 BW samples sent for MC&S had positive microbial culture, similar toe findings in a South African study.^12^

Investigating interstitial lung diseases (ILDs), hinges on obtaining lung tissue for histology and supported by bronchoalveolar lavage for comprehensive differential cell count and immune characteristics of the BAL fluid. Flexible bronchoscopy has been the investigation of choice for most ILDs including pulmonary sarcoidosis and the diagnostic methods employed are transbronchial biopsy (TBB), endobronchial biopsy (EBB), endobronchial ultrasound-guided transbronchial needle aspiration (EBUS-TBNA) and bronchoalveolar lavage.^22^ Transbronchial biopsy compared to the other methods, has shown a higher diagnostic yield (≥80%) in sarcoidosis particularly in those with significant parenchymal lesions (stage 11 disease and above) and this diagnostic accuracy is further enhanced when combined with endobronchial mucosal biopsy.^23^,^24^ This procedure requires adequate expertise, which is lacking in our unit, due to the increased risk of developing a pneumothorax in inexperienced hands. Although EBB could have been performed in our cases, its diagnostic accuracy is highly variable from studies (40–70%) and much lower (30%) in patients with visualized normal bronchial mucosa as seen in our patients with suspected sarcoidosis

According to the joint American Thoracic Society (ATS)/ European Respiratory Society (ERS)/World Association of Sarcoidosis and other granulomatous disorders (WASOG) consensus statement, EEB alone is not recommended as a sole diagnostic sampling method in sarcoidosis but rather as an additive method.^22^,^23^,^24^ Sampled BAL fluid for differential cell count could not secure the diagnosis in our cases since the laboratory set up was not able to analyze immune characteristics of the BAL fluid. In pulmonary sarcoidosis, bronchial lavage is characterized by an increased lymphocyte count with a high CD4/CD8 ratio.^25^

On hindsight, EBB could still have been performed for the sarcoid patients despite normal bronchoscopy findings which could have improved the diagnostic yield. Clearly, the absence of TBB may have impacted the study results.

Aside the use of topical anesthetic agents like Lidocaine, current guidelines strongly recommend offering conscious sedation as pre-medication to patients undergoing bronchoscopy in the absence of contraindications, unless patient otherwise refuses.^7^,^26^ This according to the consensus statement by the American college of chest physicians, improves patient's satisfaction and tolerance of the procedure with insignificant differences in complications when compared to no sedation.^26^ In addition the physicians' examination of the airway becomes thorough when procedure is well tolerated by patient.^27^ Similarly, in a post-bronchoscopy survey in South Africa, 82% of the scoped patients described the procedure as tolerable with sedation and were willing to repeat test if necessary.^28^ The preferred drug for sedation is IV Midazolam due to its rapid onset of action and the ability to scale up dose during the procedure to a maximum dose of 5mg.^7^,^26^

In conformity to current guidelines, 97.6% of our cases received IV midazolam with an average dose of 1–2mg. Current evidence has shown that combining midazolam and a short acting opioid such as fentanyl exhibits synergistic effect that further improves patients' tolerance (especially when biopsy is required), subdues the distressing heightened cough reflex as well as the increased sympathetic effect of bronchoscopy with its attendant tachycardia.^26^,^29^ Although, recommended as a better sedation option, only 25% of our cases had this combination (Fentanyl dose range of 25–50mcg). This study, however, did not assess the patients' experience of the procedure. Atropine use in bronchoscopy as pre-medication to reduce airway secretions and prevent vasovagal reactions has been for years. These benefits have not been significantly substantiated in recent studies particularly in patients who received IV midazolam but rather exhibited a tendency towards inducing haemodynamic challenges during bronchoscopy.^30^,^31^,^32^ Atropine use is therefore no longer recommended by international guidelines,^7^,^26^ We observed that 26% of our bronchoscopy patients received Atropine as pre-medication. Again, this study is unable to comment on benefits or otherwise in these patients.

Complications related to flexible bronchoscopy has been variable (0.5–11%) but showing a downward trend in recent times compared to decades ago with a mortality risk of < 0.1%.^10^,^12^,^33^, ^34^,^35^ In spite of this safety profile, severe complications could occur especially if laid down precautions are breached and these may be classified as mechanical (directly related to procedure) or systemic, related to medications used during bronchoscopy.^36^

Mechanical complications include naso/oropharngeal trauma, vocal cords and airway trauma, bronchospasm/laryngospasm, haemorrhage, pulmonary atelectasis and pneumothorax.^34^ In our current review, 2(2.4%) of the patients (non-asthmatics) had severe bronchospasm similar to the prevalence of 2% in a study by Dreisin et al.^37^ Both cases however, resolved with nebulized bronchodilators without sequelae although the procedure was aborted altogether. There is the suggestion that sedation with Opiods can reduce episodes of broncho/laryngospasms during bronchoscopy.^38^ Interestingly, both cases received only midazolam as premedication which may have contributed to these episodes. Systemic complications such as vasovagal syncope, hypoxemia, nausea/vomiting, arrhythmias, myocardial ischemic events and death did not occur among our cases (0%). Fatal hypoxaemias are now rare due to the provision of supplementary oxygen routinely during bronchoscopy.^7^

Increased risk of bleeding has been associated with FFB especially after endobronchial biopsy of a large or well vascularized tumor and may also occur with some histological subtypes of lung cancer.^39^,^40^ There seems to be no definite consensus on the severity classification of bleeding during bronchoscopy but some studies have defined it as blood loss > 40mls within 15mins or > 50mls, duration unspecified.^35^,^36^ In the current study 3(3.5%) patients had bleeding episodes without record of the estimated amount of blood loss but the need for intervention with ice-cold normal saline and 2mls of instilled 1:10,000 adrenalin solution put this as a complication based our study definition. The incidence of post-biopsy bleeding was higher compared to other studies^33^,^35^ but arguably so because 2 of the 3 reported cases had atypical bronchial carcinoids on histology. Typically, bronchial carcinoids are significantly vascularized tumors with an estimated biopsy bleeding risk of as high as 26%.^39^,^41^ The overall complication rate was 5.9%, higher compared to the findings in Nigeria and South Africa.^10^,^12^

## Conclusion

The results from the current review have been encouraging based on the high diagnostic yield of flexible bronchoscopy, particularly for lung malignancies and GeneXpert negative PTB, and an overall minor complication rate of 5.9% without mortality. There is however, the need to widen the diagnostic scope of FFB to improve the diagnosis of ILDs in the tertiary hospital and initiate therapeutic interventions through skill set enhancement. A revised operating protocol is needed at the unit to reflect current international evidence-based practice guidelines.

### Study Limitation

This study being retrospective, used manually archived patient data hence limited by a mixture of missing and ineligible information leading to exclusion of some bronchoscopy reports from the analysis.
